# Methylene tetrahydrofolate reductase gene mutation together with anticardiolipin antibody during pregnancy: a case report

**DOI:** 10.1590/S1516-31802002000500006

**Published:** 2002-09-02

**Authors:** Egle Couto, Ricardo Barini, Marcelo Luís Nomura, Joyce Maria Annichino-Bizzacchi

**Keywords:** Anticardiolipin antibodies, Tetrahydrofolate, Reductase, Thrombosis, Antiphospholipid syndrome, Anticorpo, Anticardiolipina, Metileno, Tetrahidrofolato, Redutase, Trom-bose, Síndrome antifosfolípide

## Abstract

**CONTEXT:**

High plasmatic homocysteine levels have been associated with arterial and venous thrombosis. The C677T methylene tetrahydrofolate reductase (MTHFR) gene mutation is one of the known causes for high homocysteine levels in plasma. Anticardiolipin antibody (ACA) is also associated with thrombosis and, along with other clinical complications such as recurrent abortion and stillbirth, is part of the antiphospholipid syndrome.

**DESIGN:**

Case report.

**CASE REPORT:**

A 19-year-old woman with two gestations and one parity (G2P1) had exhibited deep venous thrombosis in her previous puerperal period. Investigation of thrombophilic factors revealed ACA-IgM and heterozygous C677T mutation in the MTHFR gene. Lupus anticoagulant, protein C, protein S and antithrombin III deficiencies, and Leiden factor V and the G20210A mutation in the prothrombin gene, were not detected. The patient received 55,000 IU of subcutaneous heparin daily, from the 15 to the 36 week of pregnancy, when vaginal delivery took place. There were no clinical complications during the puerperal period and she was discharged three days after delivery, while still using oral anticoagulants.

## RESUMO

**CONTEXTO:**

A presença de níveis plasmáticos elevados da homocisteína tem sido associada à ocorrência de trombose arterial e venosa. Uma das causas da elevação da homocisteína plasmática é a presença da mutação C677T no gene da enzima metilenotetrahidrofolato redutase (MTHFR). O anticorpo anticardiolipina (ACA) é associado à ocorrência de trombose e faz parte da síndrome antifosfolípide, juntamente com outras complicações clínicas, como aborto recorrente e óbito fetal.

**TIPO DE ESTUDO:**

Relato de caso.

**RELATO DE CASO:**

Secundigesta, 19 anos, com antecedente de trombose venosa profunda no puerpério anterior. A investigação de fatores trombofílicos revelou a presença do ACAIgM e da mutação homozigota C677Tno gene da MTHFR. Foram administradas 55.000 UI/dia de heparina subcutânea, entre a 15^a^ e a 36^a^ semanas de gestação, quando evoluiu espontaneamente para parto vaginal. O puerpério transcorreu sem intercorrências e a paciente recebeu alta hospitalar no terceiro dia pós-parto, mas sob tratamento com anticoagulante oral.

## INTRODUCTION

Anticardiolipin antibody (ACA) is an acquired thrombophilic factor directed against negatively charged phospholipids. It is one of the components of antiphospholipid syndrome, along with other clinical and obstetric complications such as thrombosis, recurrent abortion and stillbirth.^1^

High homocysteine plasmatic levels have been associated with arterial and venous thrombosis. Homocysteine is a metabolic derivative from methionine. The C677T mutation in the methylene tetrahydrofolate reductase (MTHFR) gene is one of the causes of elevated homocysteine levels in plasma and is responsible for a thermolabile variant of the enzyme, with reduced activity.^2^

We report on a pregnant woman who had had deep venous thrombosis in her previous puerperal period. The investigation of thrombophilic factors included lupus anticoagulant, protein C, protein S and antithrombin III deficiencies, and Leiden factor V and the G20210A mutation, all of them with negative results. Anticardiolipin antibody and C677T homozygous mutation in the MTHFR gene were the thrombophilic factors detected in this patient.

## CASE REPORT

P.A.M., a 19-year-old woman with two gestations and one parity (G2P1) had presented deep venous thrombosis and pulmonary embolism in her previous puerperal period. Nine months later, when using oral contraceptives, she presented the same clinical features. During that pregnancy, thrombophilic factors were investigated, including acquired factors (anticardiolipin antibody and lupus anticoagulant) and inherited ones (protein C, protein S and antithrombin III deficiencies, the C677T mutation in the MTHFR gene, Leiden factor V and the G20210A mutation in the prothrombin gene).

ACA was detected using ELISA^1^ (Enzyme Linked Immunosorbent Assay), and lupus anticoagulant was detected using dilute Russell Viper Venom Time^3^ (dRVVT). Protein C, protein S and antithrombin III deficiencies were evaluated using coagulometric and chromogenic methods.^2^ DNA was amplified using the Polymerase Chain Reaction (PCR) to study the mutations Leiden factor V, G20210A in the prothrombin gene and C677T in the MTHFR gene.^2^

There were positive findings of ACA-IgM and the homozygous C677T mutation in the MTHFR gene.

Heparin was administered subcutaneously, at 55,000 IU per day from the 15^th^ to the 36^th^ week, when vaginal delivery took place. There were no complications during the puerperal period and the patient was discharged after three days, while still using oral anticoagulants.

## DISCUSSION

We have described a pregnant woman who had both acquired (ACA) and inherited (C677T in the MTHFR gene) thrombophilic factors. Prior to the pregnancy in question, she had had two episodes of thrombosis when exposed to risk situations (pregnancy and use of oral contraceptives).

ACA is one of the antiphospholipid antibodies, which are immunoglobulins directed against phospholipids with negatively charged membranes. Thrombosis and fetal distress have been associated with the presence of ACA, and it has been suggested that this antibody could react with placental antigens and inhibit placental growth and nutrient transport.^1^

The use of oral contraceptives by women with thrombogenic factors can lead to complications. A “catastrophic antiphospholipid syndrome” has been reported, with multiple organ failure, in 31 women with antiphospholipid antibodies. Situations associated with higher risks for thrombosis were identified in one third of these women, including the use of oral contraceptives.^4^

The C677T MTHFR mutation has been described as being responsible for moderate hyperhomocysteinemia. This mutation leads to a thermolabile enzyme with 50% of normal activity, which in turn explains the rise in plasma homocysteine. Homocysteine is an amino acid produced by metabolic conversion of methionine. Inherited or acquired conditions such as dietetic folate deficiency or reduced enzymatic activity can lead to hyperhomocysteinemia. A rise in homocysteine levels has been considered to be an important risk factor for arterial disease or deep venous thrombosis in the general population, even in young individuals.^2^

Comparing women with uncomplicated pregnancies with those presenting at least one of the gestational complications of severe preeclampsia, abruptio placentae, intrauterine growth restriction and stillbirths, it was observed that the presence of the C677T MTHFR homozygotic gene mutation was statistically significant in the latter group.^5^

It is well established that multiple factors interact for thrombosis onset. Several acquired and inherited factors must act together to overcome the potent natural antithrombotic mechanisms, but the interaction of these factors is poorly understood. It is important to emphasize that there are no reports of associations between these two thrombogenic factors and their specific etiologies. It must be stressed that ACA is an acquired condition and the MTHFR gene mutation is inherited. There are no reports in the literature suggesting that the presence of ACA could be induced by the MTHFR mutation.

The patient described in this report presented significant risk factors for thromboembolic and obstetric complications. However, the identification of these risk factors associated with appropriate therapy led to a good outcome for both mother and newborn. We suggest that patients with previous episodes of venous thrombosis should be screened for antiphospholipid syndrome and other inherited thrombophilic factors, so that their real risk for recurrent thromboembolism and pregnancy complications can be established.
